# Detecting Early Warning Signal of Influenza A Disease Using Sample-Specific Dynamical Network Biomarkers

**DOI:** 10.1155/2018/6807059

**Published:** 2018-01-31

**Authors:** Shanshan Zhu, Jie Gao, Tao Ding, Junhua Xu, Min Wu

**Affiliations:** School of Science, Jiangnan University, Wuxi 214122, China

## Abstract

*Aims/Introduction*. Evidences have shown that the deteriorated procession of disease is not a smooth change with time and conditions, in which a critical transition point denoted as predisease state drives the state from normal to disease. Considering individual differences, this paper provides a sample-specific method that constructs an index with individual-specific dynamical network biomarkers (DNB) which are defined as early warning index (EWI) for detecting predisease state of individual sample. Based on microarray data of influenza A disease, 144 genes are selected as DNB and the 7th time period is defined as predisease state. In addition, according to functional analysis of the discovered DNB, it is relevant with experience data, which can illustrate the effectiveness of our sample-specific method.

## 1. Introduction

A drastic change in the complex biological processes has been shown in recent studies, after which the system shifts rapidly from a stable state to another [1, 2]. This tipping point may be better known with respect to the earth climate system [3] and global finance [4] but now is applied in other areas gradually such as complex disease [5, 6]. In present studies, the disease progression is divided into three parts named normal state, predisease state (tipping point), and disease state, respectively [7]. Researches on predisease state of complex disease are only used to provide clinical patients with disease state the necessary information and are not to predict a patient in predisease state directly.

The earliest disease progression is identified by using a single molecular biomarker [8]. With further researches on disease progression, as early as 2008, Jin et al. applied the protein network to cardiovascular diseases, by identifying a group with high confidence of interacting proteins to form a network, which can be more accurate to divide into two groups of patients compared with a single molecular biomarker [9]. A more important role of network markers and a single molecular biomarker is to distinguish disease status, rather than to detect the critical state of the disease. Given this situation, Chen et al. proposed a theory of DNB to identify the critical state of the disease, which was based on model free, small sample, and high-throughput data. Three conditions to determine the DNB are put forward [10]. Generally, the studies of identifying predisease state are based on two types of data (high-throughput data and sequence data). Gao et al. extracted the larger mutation of influenza A virus proteins to form DNB based on sequence data with consecutive years, and according to the changes in DNB of each year, a warning index can be constructed to identify the outbreak year and before [11]. In addition, the development of high-throughput technology enables us to observe a large number of biomolecules by one time. Even if the number of patients' samples in the early state is small, it can also maintain each sampling point with high-throughput data on molecular level of high dimensionality [12–15].

Based on rapid advanced high-throughput technologies, we can obtain gene or protein expression at genome-wild scale with over thousands of measurements of long-term dynamics. Considering individual differences, our study is different from the method with multiple patient samples at each time period for detecting predisease state instead of proposing a sample-specific method [16–18]. In our study, the data sets which are divided into case group and control group were used to select differential expressed genes (DEGs) by *t*-test. Genes in DEGs were clustered into 40 categories by using hierarchical clustering analysis. Then, 144 genes in a group which satisfied the three criteria of DNB identification proposed by Chen were selected as DNB. Therefore, based on individual-specific data, we can predict and identify whether a time period is in predisease state by observing the variation of EWI value combined with the three indicators.

## 2. Materials and Methods

### 2.1. Data Collection

The microarray gene expression data is downloaded from the National Center for Biotechnology Information's Gene Expression Omnibus (GEO) database (series accession number: GSE19392). The gene expression data set is generated by using Affymetrix HT Human Genome U133A (HT_ HG-U133A) Microarrays, which are obtained from an experiment of primary human bronchial epithelial cells that are infected with the wild-type PR8 influenza virus (A/PR/8/34). In our study, 10 out of 20 samples are defined as case group which are collected from primary human bronchial epithelial cells infected with the wild-type PR8 influenza virus after 0.25 h, 0.5 h, 1 h, 1.5 h, 2 h, 4 h, 6 h, 8 h, 12 h, and 18 h and the rest of the 20 samples are defined as control group which treated the same process but in the absence of virus. Moreover, 22277 probe sets are mapped to 13915 unique gene symbols involved in the influenza data set.

The Student *t*-test, which can evaluate the significance of genes with differential expression between case group and control group, is applied in the selection of DEGs. The *p* value figured out by *t*-test is directly used for the subsequent filtering analysis without multiple-testing correction. Only the genes with *p* < 0.05 are regarded as DEGs.

#### 2.1.1. Dynamical System Model

Studies have shown that a biological process of the complex disease can be divided into three parts concretely. The state between normal and disease state is a tipping point which called predisease state. The system will change dramatically when the phase of disease approaches to the state. The following discrete-time state system of a living organism can be described by a nonlinear dynamical system equation:

(1) $$\begin{array}{c} A\left(\;t+1\;\right)=f\left(\;A\left(\;t\;\right);p\;\right), \end{array}$$

where *A*(*t*) = (*A*_1_(*t*),…,*A*_*n*_(*t*))′ is an *n-*dimensional vector which represents observed values or molecule concentrations (e.g., gene expression or protein expression) at time point *t*  (*t* = 0,1,…), for example, minutes, hours, or days. Parameter *P* = (*p*_1_,…, *p*_*s*_) indicates the slowly changing factors about genetic factors (e.g., SNP and CNV) and epigenetic factors (e.g., methylation and acetylation). Yet *p* is determined by its character and is not taken into consideration in this study because it is a unknown parameter with slower dynamics than *A*(*t*). *f* is general nonlinear functions of *A*(*t*).

#### 2.1.2. Data Normalization

The observed values or molecule concentrations *A*(*t*) can be classified into two groups, namely, the case group and the control group. They are denoted as *A*_case_(*t*) and *A*_control_(*t*), respectively:

(2) Acaset=A1caset,…,Ancaset′,Acontrolt=A1controlt,…,Ancontrolt′.

Due to the existing large differences in the expression values of various genes or proteins, the data normalization manner as follows is adopted to analyze the data:

(3) $$\begin{array}{c} {\hat{a}}_{nt}=\frac{A_{n}^{case}\left(t\right)-mean\left(\sum_{t}A_{n}^{control}\left(t\right)\right)}{SD\left(\sum_{t}A_{n}^{control}\left(t\right)\right)}, \end{array}$$

where *A*_*n*_^case^(*t*) is the *n*th gene or protein expression of case group and mean(∑_*t*_ *A*_*n*_^control^(*t*)) and SD(∑_*t*_ *A*_*n*_^control^(*t*)) are the mean and standard deviation of *n*th gene or protein expression at all time points in control group and the control group, respectively. Then a *n* × *t* normalization matrix is obtained:

(4) $$\begin{array}{c} \tilde{A}=\left[\begin{array}{cccc} {\tilde{a}}_{11} & {\tilde{a}}_{12} & \cdots & {\tilde{a}}_{1t}\\ {\tilde{a}}_{21} & {\tilde{z}}_{22} & \cdots & {\tilde{a}}_{2t}\\ \vdots & \vdots & \vdots & \vdots \\ {\tilde{a}}_{n1} & {\tilde{a}}_{n2} & \cdots & {\tilde{a}}_{nt} \end{array}\right], \end{array}$$

where ${\tilde{a}}_{nt}$ represents the normalization data of the *n*th gene or protein at time point* t*.

#### 2.1.3. Sample-Specific Dynamical Network Biomarkers Selection

To further filtrate DNB, DEGs by *t*-test are isolated from normalization data $\tilde{A}$ and denoted as ${\tilde{A}}_{1}$, while the rest are denoted as ${\tilde{A}}_{2}$. It is assumed that ${\tilde{A}}_{1}$ and ${\tilde{A}}_{2}$ are *r* × *t* matrix and (*n* − *r*) × *t* matrix, respectively. And ${\tilde{A}}_{1}$ is clustered into 40 categories by using hierarchical clustering analysis. The Euclidean distance is applied to calculate the distance within genes or proteins of ${\tilde{A}}_{1}$. The optimal group of genes or proteins is selected as DNB according to the following three criteria of DNB identification proposed by Chen:

(i) The average standard deviation (SD) of molecule concentration $\left({\tilde{A}}_{i}\right)$ in this group is significantly higher comparing to others.

(ii) The average Pearson correlation coefficient (PCC) in absolute value of molecule concentrations $\left({\tilde{A}}_{i}\right)$ in this group is relatively higher than the PCC between other molecules.

(iii) The average Pearson correlation coefficient in absolute value between molecule concentrations inside this group $\left({\tilde{A}}_{i}\right)$ and anyone outside this group $\left({\tilde{A}}_{j}\right)$ (OPCC) is much lower.

#### 2.1.4. Construct Sample-Specific Early Warning Index

**Figure 1:**
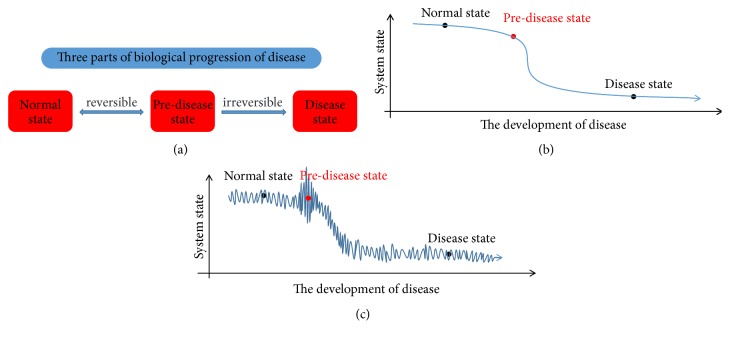
Three parts of biological progression of disease of a living organism. (a) The disease progression consists of three states including normal state, predisease state, and disease state, respectively. As shown in the picture, the process from normal to predisease state is reversible, whereas the process from predisease to disease state is irreversible. (b) The static variation displays the development progression of disease and the average value of molecule concentrations (e.g., gene or protein expression) at each state. (c) The dynamic variation shows the development progression of disease and the dynamic value of molecule concentrations (e.g., gene or protein expression) at each state.

The optimal group containing *q* genes or proteins is separated from ${\tilde{A}}_{1}$, which is marked as ${\tilde{A}}_{DNB}$. Additionally, the rest of the groups of $\tilde{A}$ are assigned to ${\tilde{A}}_{other}$. There is a key point called predisease state during the development of the disease, in the figure of dynamic state (Figure 1(c)) originally proposed by Chen et al. [10]. The change of DNB before and after predisease state is relatively stable and smooth, whereas it turns into abrupt and drastic at predisease state. After identifying the DNB, the early warning index of each time point *t* can be constructed by three criteria:

(i) The average coefficient variation (CV) of molecule concentrations at different time points is the value of fluctuation. The CV value approaching predisease state is higher than that of other time point.

(ii) The average value of absolute difference (DIF) in molecule concentrations inside DNB approaching predisease state drastically decreases compared with the values at other time points.

(iii) The average value of absolute difference between molecule concentrations inside DNB and any other outside DNB (ODIF) approaching predisease state is relatively higher than others.

Hence, the EWI_*t*_ of all time points can be constructed as

(5) $$\begin{array}{c} {EWI}_{t}=\frac{{CV}_{t}\times {DIF}_{t}}{{ODIF}_{t}}, \end{array}$$

where

(6) CVt=SDA~DNBtmeanA~DNBt

(7) DIFt=∑i1,i2a~i1t−a~i2ti1×i2i1,i2=1,2,…,  the  number  of  DNB

(8) ODIFt=∑i,ja~it−a~jti×jj=1,2,…,  the  number  of  genes  or  proteins  outside  DNB.

In the light of the characteristic in predisease state, a time point with the largest value can be considered as the predisease state. After the point, the disease progression of a living organism shifts rapidly from normal to disease state.

## 3. Result

All without-correct-corresponding gene symbols are screened out, and probe of the same genes is combined by the averaging method. There are 13915 genes left. Based on the 13915 genes, Student's *t*-test is applied to calculate the *p* value of each gene by comparing its expression profile between case groups and control groups. We identify 264 genes with *p* < 0.05 as DEGs. Next, 264 genes are classified by hierarchical clustering analysis into 40 categories. Analyzing all clusters or groups, a group of 144 genes is identified as DNB, which satisfies the three criteria of DNB identification. Among them, the values of average SD and average PCC in this group are 1.2585797 and 0.3047569, which is higher than others (e.g., 0.8802955 and 0.2940955), and the average OPCC is relatively high.

**Figure 2:**
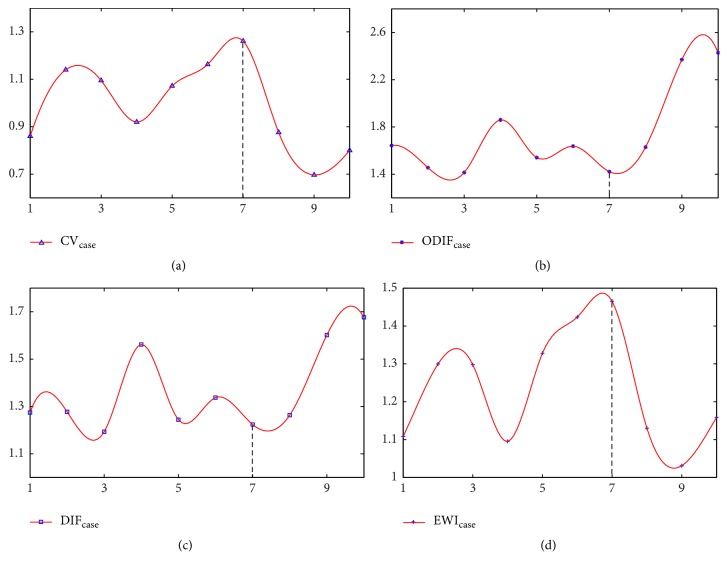
The early warning index of influenza disease. In all the figures, the abscissa represents the time point* t.* (a) The average coefficient variation (CV) of genes expression in DNB at 10 time points. (b) The average difference in absolute value between genes expression inside DNB and any other outside DNB (ODIF) at 10 time points. (c) The average difference (DIF) in absolute value of genes expression in DNB at 10 time points. (d) The early warning index (EWI) of the case set of high-throughput experimental data for influenza A disease.

To further clarify the early warning index for influenza A disease with 10 time points, Figure 2 demonstrates variation of four indicators in detail. As shown in (a), the curve of the CV value of DNB strongly fluctuates with time and the value at 7th time point (6 h) reaches the maximum value, which indicates that the genes in DNB change drastically when approaching 7th time point (6 h). And the value of DIF at 6 h shows the relatively lower value, which indicates that the trend of genes expression in DNB is similarly approaching 7th time point (6 h). Although the change in ODIF is not obvious, the early warning index at 7th time point (6 h) reaches the maximum value. Thus, the most prominent physiological effects occur approaching 7th time point (6 h). Meanwhile, gene expression changes in HBECs in response to wild-type influenza (PR8) show a strong float after 7th time point (6 h) [19].

In order to analyze biological functional of the obtained DNB, A bioinformatics database DAVID [20] (https://david.abcc.ncifcrf.gov/) with Gene Ontology (GO) analysis and KEGG Pathway analysis is mentioned. Some enriched GO functions based on identified genes in DNB are listed in Table 1. Gene Ontology can be divided into three parts: molecular function, biological process, and cellular composition. The analysis of genes reveals that the DNB selected by the sample-specific method is particularly related to influenza disease, which confirms the validation of our theory about the increasing index approaching predisease state. The enriched GO functions underlying the identified DNBs are particularly related to immune systems that are activated to protect against influenza A virus and inordinate dysfunctions associated with the performance in the viral life cycle. In the DNBs, DCAF1 and ADGRG3, which play crucial roles in cell differentiation, and ARVCF, COL19A1, and OLR1, which are associated with cell adhesion, regulate the expression of cell adhesion molecules [21]. Further, some genes in the basic cellular processes are expressed in a disorderly manner, for example, IFNA10, which is associated with the regulation of cell death and abnormal reaction in transcription and translation. Moreover, Some of them are involved in the related triglyceride metabolic process, especially for APOC3.

According to KEGG Pathway enrichment analysis, the results show that genes in DNB of influenza A disease are closely relevant to immune system and inflammation, such as cytokine-cytokine receptor interaction, PPAR signaling pathway, and Jak-STAT signaling pathway in Table 2. As key genes in cytokine-cytokine receptor interaction, CXCR6, IFNA10, IL21R, and TGFB3 in DNB participate in immune response and immune regulation, regulate innate immune and adapt immune response, stimulate hematopoietic function, stimulate cell activation, proliferation, and differentiation, and induce apoptosis. The pathway of cytokine-cytokine receptor interaction is the same with expressed data.

**Figure 3:**
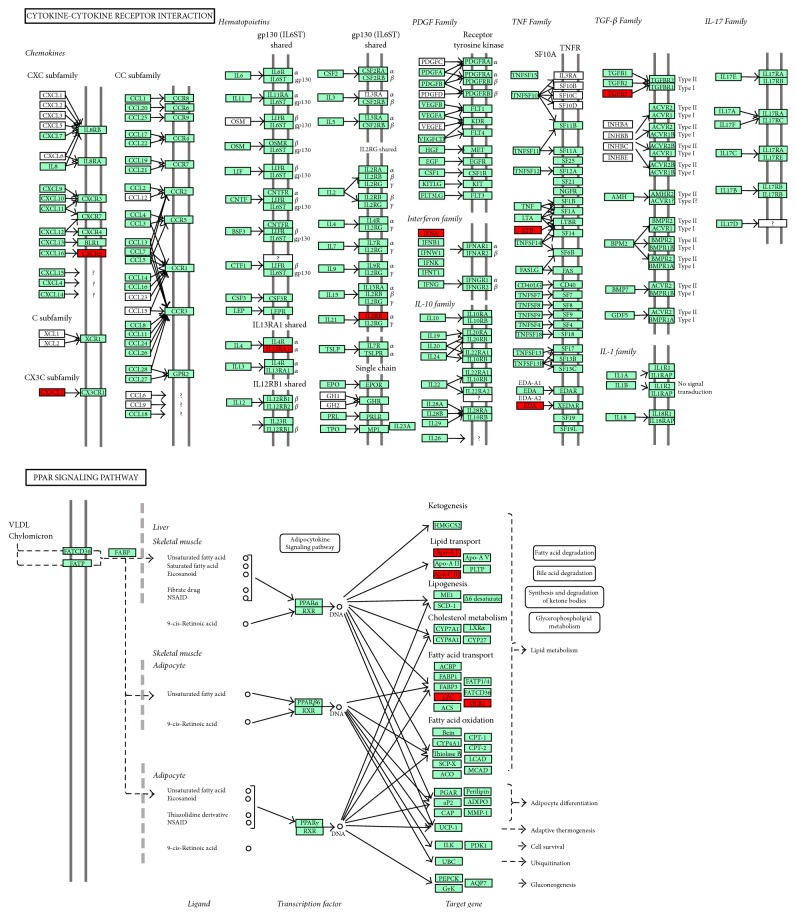
Key biological pathways with DNB genes in cytokine-cytokine receptor interaction and PPAR signaling pathway.

Moreover, the genes in PPAR signaling pathway like LPL, APOA1, OLR1, and APOC3 play a significant role in inhibiting inflammation, regulating cell apoptosis and immune system. And the genes in DNB which are marked red are placed the critical positions in cytokine-cytokine receptor interaction and PPAR signaling pathway. As shown in Figure 3. JAK-STAT signaling pathway is a signal transduction pathway stimulated by cytokines in recent years [22], which includes IFNA10, IL21R, IL13RA1, and IL3RA in DNB, involved in cell proliferation, differentiation, apoptosis, and immune regulation, and many other important biological processes.

To further demonstrate the effectiveness of our method, we analyze symptoms of patients and their complications. Patients develop symptoms of illness of upper respiratory tract infection. However, they are also accompanied by the occurrence of pulmonary complications, and renal failure [23]. Moreover, 18 out of 144 genes are validated with significantly close relation with influenza A disease. The CX3CL1 involves both acute and chronic inflammations, which is characterized by major perturbations of the immune homeostasis [24]. Especially surfactant proteins SFTPB plays a key role in alveolar stability [25], Which is associated with influenza A disease. And this gene encodes the pulmonary-associated surfactant protein B. The surfactant is secreted by the alveolar cells of the lung and maintains the stability of pulmonary tissue by reducing the surface tension of fluids that coat the lung.

## 4. Discussion

To detect the early warning signal of influenza A disease using a small number of samples of high-throughput data, we propose an early warning index serving as a leading indicator to predict the critical transition based on the concept of dynamical network biomarkers proposed by Chen, which drives the disease progression from normal state to disease state. Compared to the general biomarkers [26], dynamical network biomarkers are more suitable for characterizing the transfer of system status. In our study, We first select the DEGs by *t*-test between case groups and control groups. Then, a new type of normalization data is constructed by the formula defined in this study for the sake of analysis of the next step. Different from the previous methods, our work regards the gene expression with time of each gene as a vector for hierarchical clustering analysis. And the Euclidean distance is applied to calculate the distance within genes in DEGs. A group, which satisfies three criteria of DNB identification, is identified as DNB. Further, the values of CV, DIF, and ODIF are calculated to construct an index for detecting predisease state of individual sample. The index EWI is applied in early diagnosis with the microarray data of influenza A disease, which demonstrates fluctuated values with time. Although the ODIF value approaching predisease state is not completely obvious, the expression value of the other three indicators is significantly relevant with our theory. In addition, everyone with the same disease has different DNB due to different driving factors. We will focus on this important future topic and continue to refine the algorithm in later research.

## Figures and Tables

**Table 1:** Functional enrichment of GO for part of genes of identified DNB.

GO term	Description	DNB	*p* value	Corrected *p* value
GO:0007155	Cell adhesion	ARVCF, COL19A1, OLR1, CD300A, PCDHA10, CX3CL1, OMG, SIGLEC9	0.03844	0.85044
GO:0045766	Positive regulation of angiogenesis	ADM2, NOS3, CX3CL1, ALOX12	0.04448	0.86098
GO:0005886	Plasma membrane	SCN3B, GRIK2, TGFB3, SLC7A9, DMPK, APOA1,…	9.55*E* − 7	1.56*E* − 4
GO:0005615	Extracellular space	LPL, CRISP3, LUM, AFM, IFNA10, TGFB3, CX3CL, BMP15, APOA1, APOL1, IL18BP,…	1.23*E* − 4	0.00400
GO:0005102	Receptor binding	HAO1, LPL, TENM2, HAO2, CX3CL1, LTB	0.03689	0.97736
GO:0010181	FMN binding	HAO1, HAO2, NOS3	0.00418	0.67592

**Table 2:** Functional enrichment of KEGG pathways for part of genes of identified DNB.

Pathway term		DNB	*p* value
hsa04060	Cytokine-cytokine receptor interaction	CXCR6; IFNA10; IL21R; TGFB3; LTB; EDA2R; CX3CL1; IL13RA1; IL3RA	3.24*E* − 4
hsa03320	PPAR signaling pathway	LPL; APOA1; OLR1; APOC3	1.45*E* − 2

## References

[1] Joost Lesterhuis W., Bosco A., Millward M. J., Small M., Nowak A. K., Lake R. A. (2017). Dynamic versus static biomarkers in cancer immune checkpoint blockade: Unravelling complexity. *Nature Reviews Drug Discovery*.

[2] Dahlem M. A., Kurths J., Ferrari M. D., Aihara K., Scheffer M., May A. (2015). Understanding migraine using dynamic network biomarkers. *Cephalalgia*.

[3] Nikolaou A., Gutiérrez P. A., Durán A., Dicaire I., Fernández-Navarro F., Hervás-Martínez C. (2015). Detection of early warning signals in paleoclimate data using a genetic time series segmentation algorithm. *Climate Dynamics*.

[4] Karahoca D., Karahoca A., Yavuz Ö. (2013). An early warning system approach for the identification of currency crises with data mining techniques. *Neural Computing and Applications*.

[5] Liu R., Li M., Liu Z.-P., Wu J., Chen L., Aihara K. (2012). Identifying critical transitions and their leading biomolecular networks in complex diseases. *Scientific Reports*.

[6] Chen P., Liu R., Li Y., Chen L. (2016). Detecting critical state before phase transition of complex biological systems by hidden Markov model. *Bioinformatics*.

[7] Achiron A., Grotto I., Balicer R., Magalashvili D., Feldman A., Gurevich M. (2010). Microarray analysis identifies altered regulation of nuclear receptor family members in the pre-disease state of multiple sclerosis. *Neurobiology of Disease*.

[8] Kinney J. S., Morelli T., Braun T. (2011). Saliva/pathogen biomarker signatures and periodontal disease progression. *Journal of Dental Research*.

[9] Jin G., Zhou X., Wang H. (2008). The knowledge-integrated network biomarkers discovery for major adverse cardiac events. *Journal of Proteome Research*.

[10] Chen L., Liu R., Liu Z.-P., Li M., Aihara K. (2012). Detecting early-warning signals for sudden deterioration of complex diseases by dynamical network biomarkers. *Scientific Reports*.

[11] Gao J., Zhang L., Jin P. (2014). Influenza pandemic early warning research on HA/NA protein sequences. *Current Bioinformatics*.

[12] Zeng T., Sun S.-Y., Wang Y., Zhu H., Chen L. (2013). Network biomarkers reveal dysfunctional gene regulations during disease progression. *FEBS Journal*.

[13] Liu R., Wang X., Aihara K., Chen L. (2014). Early diagnosis of complex diseases by molecular biomarkers, network biomarkers, and dynamical network biomarkers. *Medicinal Research Reviews*.

[14] Li M., Zeng T., Liu R., Chen L. (2014). Detecting tissue-specific early warning signals for complex diseases based on dynamical network biomarkers: Study of type 2 diabetes by cross-tissue analysis. *Briefings in Bioinformatics*.

[15] Torshizi A. D., Petzold L. (2017). Sparse Pathway-Induced Dynamic Network Biomarker Discovery for Early Warning Signal Detection in Complex Diseases. *IEEE/ACM Transactions on Computational Biology & Bioinformatics*.

[16] Liu X., Wang Y., Ji H., Aihara K., Chen L. (2016). Personalized characterization of diseases using sample-specific networks. *Nucleic Acids Research*.

[17] Liu X., Chang X., Liu R., Yu X., Chen L., Aihara K. (2017). Quantifying critical states of complex diseases using single-sample dynamic network biomarkers. *PLoS Computational Biology*.

[18] Liu R., Yu X., Liu X., Xu D., Aihara K., Chen L. (2014). Identifying critical transitions of complex diseases based on a single sample. *Bioinformatics*.

[19] Shapira S. D., Gat-Viks I., Shum B. O. V. (2009). A physical and regulatory map of host-influenza interactions reveals pathways in H1N1 infection. *Cell*.

[20] Sherman B. T., Huang D. W., Tan Q. (2007). DAVID Knowledgebase: a gene-centered database integrating heterogeneous gene annotation resources to facilitate high-throughput gene functional analysis. *BMC Bioinformatics*.

[21] Zhang D., Tang N., Liu Y., Wang E.-H. (2015). ARVCF expression is significantly correlated with the malignant phenotype of non-small cell lung cancer. *Molecular Carcinogenesis*.

[22] Hsu S.-F., Su W.-C., Jeng K.-S., Lai M. M. C. (2015). A host susceptibility gene, DR1, facilitates influenza a virus replication by suppressing host innate immunity and enhancing viral RNA replication. *Journal of Virology*.

[23] Antonopoulou A., Baziaka F., Tsaganos T. (2012). Role of tumor necrosis factor gene single nucleotide polymorphisms in the natural course of 2009 influenza A H1N1 virus infection. *International Journal of Infectious Diseases*.

[24] Liu W., Jiang L., Bian C. (2016). Role of CX3CL1 in Diseases. *Archivum Immunologiae et Therapia Experimentalis*.

[25] To K. K. W., Zhou J., Song Y.-Q. (2014). Surfactant protein B gene polymorphism is associated with severe influenza. *CHEST*.

[26] Liu Z.-P. (2016). Identifying network-based biomarkers of complex diseases from high-throughput data. *Biomarkers in Medicine*.

